# A text messaging intervention to improve retention in care and virologic suppression in a U.S. urban safety-net HIV clinic: study protocol for the Connect4Care (C4C) randomized controlled trial

**DOI:** 10.1186/s12879-014-0718-6

**Published:** 2014-12-31

**Authors:** Katerina A Christopoulos, Elise D Riley, Jacqueline Tulsky, Adam W Carrico, Judith T Moskowitz, Leslie Wilson, Lara S Coffin, Veesta Falahati, Jordan Akerley, Joan F Hilton

**Affiliations:** HIV/AIDS Division, San Francisco General Hospital, University of California San Francisco, 995 Potrero Avenue, 4th Floor, San Francisco, 94110 CA USA; School of Nursing, University of California San Francisco, San Francisco, CA USA; Medical Social Sciences, Feinberg School of Medicine, Northwestern University, Chicago, IL USA; School of Pharmacy, University of California San Francisco, San Francisco, CA USA; Global Health Sciences, University of California San Francisco, San Francisco, CA USA; HIV Services, The Shanti Project, San Francisco, CA USA; Department of Epidemiology and Biostatistics, University of California San Francisco, San Francisco, CA USA

**Keywords:** Short message service, SMS, Text messaging, HIV, Retention in HIV care

## Abstract

**Background:**

Few data exist on the use of text messaging as a tool to promote retention in HIV care and virologic suppression at the clinic level in the United States. We describe the protocol for a study designed to investigate whether a text messaging intervention that supports healthy behaviors, encourages consistent engagement with care, and promotes antiretroviral persistence can improve retention in care and virologic suppression among patients in an urban safety-net HIV clinic in San Francisco.

**Methods/Design:**

Connect4Care (C4C) is a single-site, randomized year-long study of text message appointment reminders vs. text message appointment reminders plus thrice-weekly supportive, informational, and motivational text messages. Eligible consenting patients are allocated 1:1 to the two arms within strata defined by HIV diagnosis within the past 12 months (i.e. “newly diagnosed”) vs. earlier. Study participants must receive primary care at the San Francisco General Hospital HIV clinic, speak English, possess a cell phone and be willing to send/receive up to 25 text messages per month, a have viral load >200 copies/μL, and be either new to the clinic or have a history of poor retention. The primary efficacy outcome is virologic suppression at 12 months and the key secondary outcome, which will also be examined as a mediator of the primary outcome, is retention in HIV care, as operationalized by kept and missed primary care visits. Process outcomes include text message response rate and percent of time in study without cell phone service. Generalized estimating equation log-binomial models will be used for intent to treat, per protocol, and mediation analyses. An assessment of the cost and cost-effectiveness of the intervention is planned along with a qualitative evaluation of the intervention.

**Discussion:**

Findings from this study will provide valuable information about the use of behavioral-theory based text messaging to promote retention in HIV care and virologic suppression, further elucidate the challenges of using texting technology with marginalized urban populations, and help guide the development of new mobile health strategies to improve HIV care cascade outcomes.

**Trial registration:**

NCT01917994

**Electronic supplementary material:**

The online version of this article (doi:10.1186/s12879-014-0718-6) contains supplementary material, which is available to authorized users.

## Background

Retention in care is a key step in the HIV treatment cascade, the aim of which is to ensure that all individuals living with HIV are successfully treated [1]. Missed primary care visits have been associated with longer time to antiretroviral initiation and shorter time to virologic failure and death [2]-[4]. In addition to the timely management of HIV infection, regular visit attendance allows other important interventions to occur, such as risk reduction counseling, mental health and substance use referrals, and health care maintenance [5],[6]. Despite the proven health and prevention benefits of consistent HIV care, only about half of individuals living with HIV in the United States are estimated to be retained in care and only one-quarter are virologically suppressed [7].

Reasons for poor retention in HIV care are diverse and span individual (e.g. depression, stigma), interpersonal (e.g. patient-provider relationship), and structural (e.g. insurance eligibility) factors [8]. Evidence-based interventions to promote retention in care and virologic suppression include adherence counseling, medical case management, intensive outreach, and peer or paraprofessional patient navigation [9]. In addition, studies have shown that prompts and reminders are effective tools for both antiretroviral and appointment adherence [10]-[12]. Moreover, the randomized, multi-site Centers for Disease Control (CDC) Retention in Care project, demonstrated that patients receiving enhanced personal contact in addition to usual clinic practice exhibited a significant improvement in appointment attendance [13].

Short message service (SMS) technology represents a promising new strategy for HIV care. SMS has been deployed successfully in support of antiretroviral adherence and virologic suppression in sub-Saharan Africa and is currently being studied in this setting with regard to retention in care [14]-[16]. In the U.S., studies to date of text messaging in HIV-infected populations have generally focused on sub-groups, such as youth, substance users, and men who have sex with men [17]-[20]. Few studies to date have examined the impact of SMS on improving retention in care and virologic suppression for broader populations of marginalized persons using safety-net HIV clinics in the U.S. In a feasibility study of 25 patients receiving SMS appointment reminders in a Southeastern U.S. clinic, challenges to receiving SMS included discomfort with cell phone use, patients not opting in to receive messages, and service interruptions [21]. It is currently unknown whether such challenges are surmountable and whether a more comprehensive SMS intervention that supports healthy behaviors, encourages consistent engagement with health care, and promotes antiretroviral persistence would significantly influence virologic suppression among marginalized patients in the U.S. In addition, examining the cost of such an intervention is an important consideration for real-world feasibility.

Connect4Care (C4C) is a randomized controlled trial (RCT) examining the efficacy of a SMS intervention to improve virologic suppression and retention in care among patients at a safety-net HIV clinic in San Francisco. The C4C SMS intervention is designed to provide information about resources for healthy living, promote intrinsic motivation for engaging in HIV care, and support enhanced psychological adjustment. This integrative approach is based on: 1) several behavioral models commonly used to understand barriers and facilitators to engagement in care [22]-[26], and; 2) a conceptual framework designed to integrate the communication functionality of SMS with psychosocial factors known to impact health outcomes [27]. C4C builds on the existing evidence base for SMS interventions that have been conducted in resource-limited settings by providing empirical data to examine SMS influences on disadvantaged individuals living in a resource-rich environment. This paper describes the C4C study protocol.

### Study objectives

#### Primary objective

To determine whether the C4C intervention improves virologic suppression at 12 months when compared to SMS appointment reminders alone.

#### Key secondary objectives

To determine whether the C4C intervention improves retention in clinic care at 12 months, as operationalized by measures of kept and missed primary care visits.

#### Other secondary objectives

1) To determine the effect of the C4C intervention on psychosocial outcomes, including reduced depressive symptomatology as well as increased positive affect, social support, health-related quality of life, and health care empowerment. 2) To assess the cost of intervention delivery and cost-effectiveness of the C4C intervention. 3) To explore participant experiences with the C4C intervention as well as barriers to and facilitators of the use of SMS technology through a qualitative sub-study.

## Methods

### Study design

**Figure 1:**
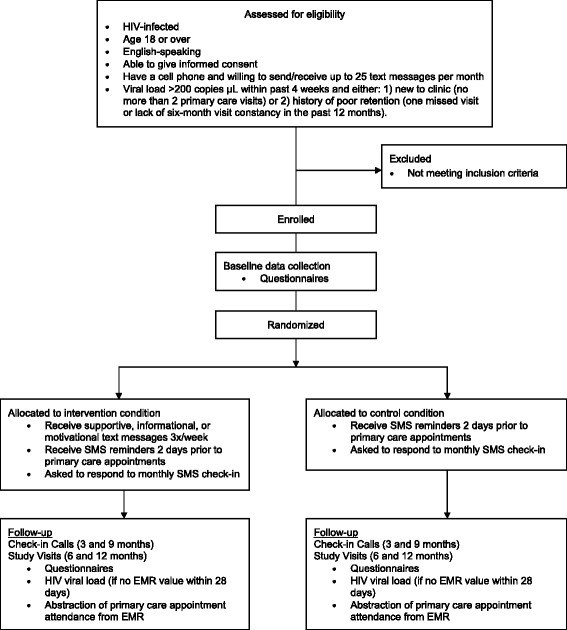
**C4C flow diagram.**

C4C is a single site, parallel arm, open-label RCT of SMS appointment reminders alone vs. SMS appointment reminders plus thrice-weekly SMS messages that deliver information and enhance motivation and psychosocial adjustment. Eligible consenting patients are allocated 1:1 to the two arms within strata defined by HIV diagnosis within the past 12 months (i.e. “newly diagnosed”) vs. earlier (Figure 1).

### Study settings

The HIV clinic at San Francisco General Hospital (SFGH), also known as Ward 86, is one of the oldest and largest public HIV clinics in the United States, serving 3,000 low-income patients as part of San Francisco County’s safety-net hospital. Patients at Ward 86 represent multiple urban populations, including the recently incarcerated, substance users, homeless and unstably housed individuals, and those with mental illness. Approximately 12% of clinic patients are women, 25% are African-American and 20% are Hispanic/Latino. Consistent with national guidelines, the clinic standard of care is to offer antiretroviral therapy to all individuals, irrespective of CD4 cell count [28]. The clinic standard of care is also to provide reminder phone calls to patients at least 2 days prior to primary care appointments, however, the consistency of this practice is dependent on staffing levels. Patients are screened for eligibility in the clinic, but baseline, 6, and 12-month study visits take place at a clinical research site in the community. This separate study site minimizes the likelihood that participation in the study influences the key secondary outcome of retention in clinic care.

### Study participants

The study sample consists of HIV-infected individuals age 18 or older who receive primary care at Ward 86, speak English, have a cell phone, can read a text message, and are willing to send or receive anywhere from 1 to 25 text messages per month. In addition, participants must have detectable viral loads (>200 copies/μL) and be either: 1) new to clinic (defined as no more than 2 primary care visits at Ward 86) or 2) have a history of poor retention (defined as one or more missed visits or lack of six-month visit constancy in the past 12 months at Ward 86).

### Inclusion criteria

- HIV-infected
- Age 18 or older
- English-speaking
- Able to give informed consent
- Have a cell phone, can read a text message, and willing to send/receive up to 25 text messages per month
- Viral load >200 copies/μL within past 4 weeks and either: 1) new to clinic (no more than 2 primary care visits at Ward 86) or 2) history of poor retention (one or more missed visits at Ward 86 or lack of six-month visit constancy in the past 12 months).

### Exclusion criteria

- Under age 18
- Non English-speaking
- Unable to give informed consent
- Viral load <200 copies/μL
- No missed visits and achievement of six-month visit constancy in the past 12 months in an individual whose viral load is >200 copies μL

#### SMS interactions with participants

Information on pending primary care appointments is downloaded from the hospital electronic medical record and transmitted automatically to a text messaging platform that sends SMS reminders two days prior to HIV primary care appointments. All study participants receive reminders stating, “You have an appointment on Tuesday, June 18th, at 11:30 am with your provider at SFGH, Building 80, 6th floor. Please call XXX-XXX-XXXX to cancel or reschedule”. Participants choose morning or afternoon times during business hours to receive appointment reminders, so that they can call the clinic to reschedule if they are unable to make the appointment.

All participants also receive a monthly text message that reads, “This is your monthly check in from C4C. Thanks for your participation. Please respond C to confirm you received this message”. If participants do not respond to the monthly check-in message, a research assistant calls the participant to ensure that the phone is working and contact information is up to date, unless the participant is in the intervention arm and noted to have responded to an intervention message in the week before or after the check-in message (see *C4C Intervention* below). The monthly check-in message was designed to promote study retention among control arm participants and to track their length of follow-up in the study, as some participants may only have primary care appointments scheduled every three to four months.

Participants are counseled on the potential for bugs and glitches in the SMS system and informed that it is not a method for communicating with study staff or with the clinic. A reply to any study SMS that does not ask for response, including the SMS appointment reminders, results in the message, “Thanks for the feedback. If this is an emergency, please call 911”. At the enrollment visit, a research assistant programs the number from which text messages are sent into the participant’s phone as “C4C Study”.

#### Control condition

The control condition includes only the SMS appointment reminders and the monthly check-in messages.

#### C4C intervention

The C4C intervention is designed to foster a sense of connectedness to one’s health and health care as a means of promoting virologic suppression and retention in care. In addition to the monthly check-in messages and appointment reminders, it consists of motivational, informational, and supportive text messages three times per week over a period of one year, based on studies showing that daily messages are less effective than weekly or less than weekly messages [14],[29]. We used a conceptual SMS framework developed by Coomes et al. [27] and drew upon several behavioral models frequently used to understand engagement in HIV care to identify important theoretical constructs [27]. These models included the Behavioral Model for Vulnerable Populations, the Information, Motivation, and Behavior Skills model, the Health Care Empowerment model, and revised Stress and Coping Theory [22]-[26],[30]. As a team of HIV clinicians and behavioral scientists, we created intervention texts in six domains (Table 1) that targeted a sixth-grade reading level. To inform the development of the intervention messages, we conducted formative research with patients and clinic staff. We conducted three focus groups with clinic social workers, linkage team members, and current clinic patients to refine the intervention content.

**Table 1:** **Intervention domains and sample text messages**

Domain	Sample text message
Improving a sense of social support	Stay strong. The clinic cares about you.
Ameliorating negative affect	Everyone feels sad sometimes. Remember you can talk to your provider about depression.
Bolstering positive affect and coping	Smile, breathe, and go slowly.
Fostering empowerment	Be active in your health care. Keep your scheduled appointments.
Supporting healthy behaviors and health maintenance	Invest in your health. Remember to get your Pap smear.
Emphasizing the value of antiretroviral adherence and persistence	Taking meds? They help, even if you can’t tell they are working.

Additional formative work included a pilot phase to test technical and administrative aspects of field operations, as well as the acceptability of intervention text messages. We obtained feedback on one month of messages from participants enrolled in an open-phase pilot (n = 10) to further refine intervention content. These pilot participants then entered an extension phase in which main study procedures and instruments were tested with this group, so as to resolve any technical glitches prior to deployment with main study participants. Pilot participant data will not be analyzed with that of randomized study participants.

Intervention messages are divided into three response types. Type 1 is a message that asks for no response (one-way). Type 2 asks participants if they found the message helpful (two-way). Type 3 asks participants if they would like more information on a topic and if they answer yes, they receive a message with additional information (three-way). Each domain has messages from each response type, and domain-specific messages are randomly ordered over the twelve-month intervention period. Intervention group participants receive at least one Type 2 or 3 message per week because we track responses to estimate the amount of intervention participants receive. Participants can choose morning, afternoon, or evening times to receive intervention messages.

### Efficacy outcomes

#### Primary outcome

The primary outcome is virologic suppression at 12 months, defined as a viral load <200 copies/μL [28].

### Key secondary outcomes

- A three-level ordinal retention variable that incorporates an assessment of *missed visits* as well as *six-month visit constancy*.
  - ○ High: Attended all scheduled primary care appointments in both 6-month study periods
  - ○ Moderate: Attended at least one scheduled clinic appointment in both 6-month study periods
  - ○ Low: Attended no scheduled clinic appointments in at least one 6-month study period
- *Visit adherence rate*, defined as the number of primary care appointments kept divided by the number of appointments scheduled, excluding cancelled or rescheduled visits.

### Other secondary outcomes

- Depression symptom severity [31]
- Positive affect [32]
- Perceived social support [33]
- Health-related quality of life [34]
- Health care empowerment [25],[35]

The rationale for assessing several measures of retention is that retention measures generally fall into two categories, those based on missed or “no-show” visits and those based on kept visits. It is deemed standard by experts in the field to use at least one measure from each category when conducting research on retention in care as these measures are thought to capture different aspects of the retention in care experience [36].

### Process outcomes

- Response rate to monthly check-in “blast”
- Response rate to Type 2 and 3 intervention messages
- Percentage of time in study without cell phone service

As is true of many randomized trials, C4C exists on the continuum between efficacy and effectiveness [37]. Efficacy studies determine whether an intervention produces the expected result under ideal circumstances and effectiveness studies measure the degree of beneficial effect in “real world” settings [38]. C4C only enrolls patients who are in clinical care and willing to participate in a research study, potentially resulting in a less generalizable sample that is more consistent with an efficacy study. However, it does not provide participants with cell phones or airtime, which acknowledges that service interruptions (and thus interruptions in the intervention) represent “real world” implementation. This study decision was based on an emerging area of importance in behavioral (non-pharmacologic) interventions [39] and text messaging studies in particular [40] – that of process evaluation, which seeks to determine whether the intervention was delivered as intended and whether participants engage with the intervention.

Participants may experience disruption in receiving SMS appointment reminders and intervention messages due to cell phones being lost or stolen, participants’ inability to pay their bills, or participant loss to follow-up. We therefore plan to track the frequency of responses to monthly check-ins for all participants and for Type 2 and 3 messages in the intervention arm. We will also record self-reported loss of phone access at study check-ins by phone and at in-person study visits.

### Challenges in intervention implementation

All C4C text messages are sent using the text messaging platform of an outside vendor. The initial text messaging vendor did not have the capacity to conduct the study as planned and thus two months into the main study the C4C team transitioned to using the open-access platform Twilio to send text messages manually until the platform of the new vendor could be deployed, which occurred six months into the main study. While enrolled participants experienced no break in study messages, we will examine the effect of vendor on efficacy during analysis.

### Data collection and management

The primary efficacy outcome of virologic suppression at 12 months will be defined by the clinic EMR closest to the study visit. Patients who do not have EMR results available within 4 weeks of the final 12-month study visit will receive phlebotomy at the study visit.

EMR data will also be used to assess the secondary efficacy outcome of retention in care. One of the shortcomings of this technique is that it measures retention in a particular clinic rather than true retention in care, which could potentially occur in multiple clinics. The EMR used in this study identifies primary care visits to other clinics in the San Francisco Department of Public Health system, which will help address this issue. If during study follow up participants indicate that they have transferred care outside of this system, we will seek their permission to obtain appointment records from the outside clinic. Scheduled and kept primary care appointments after transfer elsewhere be included in the assessment of retention; consequently, we anticipate that missing outcomes will be rare (Table 2).

**Table 2:** **Use of clinic and study data sources to ascertain study outcomes**

	Retained in C4C study	Lost to C4C study
Retained in care	Viral load and appointment data available from EMR, outside clinic records, and, when necessary, study phlebotomy	Viral load and appointment data available from EMR and (if permission obtained) outside clinic records
Not retained in care	Phlebotomy performed to collect viral load data	Tracking attempted at study end to ascertain missing outcomes

Laboratory and appointment data downloaded from clinical and hospital EMRs will be de-identified. Viral load results from study phlebotomy are transmitted to study staff in a secure spreadsheet. Baseline and follow-up questionnaires include validated psychosocial measures, including measures required by the funder’s cross-site data harmonization effort [41]. Study questionnaires also include questions about cell phone access and use. Study questionnaires are self-administered through Computer Assisted Survey Information Collection (CASIC), a web-based data collection tool, and have both computer-assisted personal interviewing (CAPI) and audio computer-assisted self-interviewing (ACASI) components. Outbound text messages and incoming responses from participants are captured in daily logs, as are failed attempts at SMS transmission to participants. A data manager merges all study data based on participant ID and performs periodic quality control for range and completeness of data. All participant data is stored on a secure password-protected server.

### Study procedures

#### Recruitment

A daily EMR query allows a research assistant to identify patients who may qualify for the study and remind providers that a particular patient may be eligible. Clinic providers introduce the study to patients and refer interested individuals to a research assistant. While explaining the study in more detail, the research assistant asks the patient to read a sample text message, enabling a brief literacy check. The research assistant assesses an individual’s visit history in the EMR and uses a checklist to complete screening with the patient. If individuals do not have viral load results or results pending in the EMR within the past four weeks, they can undergo phlebotomy through the study. Once eligibility is confirmed, individuals are scheduled to visit a community-based research site located in the Tenderloin neighborhood, where many patients reside, in order to complete informed consent, a baseline questionnaire, phone activation in the vendor platform, and randomization. To facilitate this visit, the research assistants offer same-day baseline study appointments and a bus token. Participants are reimbursed $30 cash for their time after completing baseline, 6 and 12 month study visits. They are not given phones nor are they compensated for any costs associated with the messages. If individuals are interested in the study but do not own a cell phone, research assistants refer them to the federal Assurance Wireless program, which assists low-income individuals in obtaining cell phones (http://www.assurancewireless.com/Public/Welcome.aspx).

#### Randomization

At the baseline study visit, a research assistant reviews and confirms eligibility in a study database. Following written consent, a computer program captures the enrollment time and uses unpredictable specific digits to allocate patients 1:1 to study arms. Equal allocation occurs within levels of the stratification factor (HIV diagnosis within the past 12 months vs. greater than 12 months). We stratified by this factor because the estimated virologic suppression rate for individuals newly diagnosed with HIV infection in San Francisco is higher than national averages and may exceed the rate among individuals with longer standing HIV diagnoses [42]. In addition, to maximize allocation concealment and ensure balanced allocation throughout accrual, equal allocation also occurs within randomly generated blocks of size 4, 6 or 8.

#### Blinding

As outlined by Boutron et.al., study staff cannot be blinded to intervention allocation because monitoring study participation may require follow-up phone conversations, and participants cannot be blinded because of the differential frequency and type of SMS messages by arm [39].

#### Follow-up of study participants

**Figure 2:**
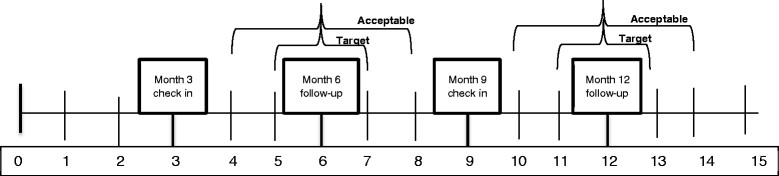
**Timeline for data collection by study month.**

Follow-up study visits occur at a community-based research site rather than in the same building as the clinic in order for the study to avoid influencing attendance at clinic visits. Both intervention and control arm participants are asked to complete 6 and 12 month study visits, at which time participants complete a self-administered questionnaire that collects secondary outcomes. If no viral load is resulted or pending in the EMR from the past 28 days then phlebotomy is performed for viral load assessment. Participants receive check-in phone calls at 3 and 9 months to remind them of the upcoming study visit and confirm that the participant’s contact information remains up to date (Figure 2).

Follow-up study visits are scheduled to occur in two-month windows around the 6 and 12 month mark [15]. However, we acknowledge the many challenges inherent in working with a marginalized urban population in a year-long study. If we are unable to contact participants but they present to the clinical research site for follow-up, we will perform data collection as outlined in Figure 2.

### Statistical methods

#### Sample size

Based on clinic data [43], we designed the trial assuming a rate of virologic suppression of 60% at 12 months in the control arm. To detect an improvement of 15% in the intervention arm with 80% power and a two-sided α = 0.05, we calculated that we would need 152 participants per arm (or, 167 participants per arm assuming 90% 12-month study retention, based on the experience of other studies using the same population). Clinic data from 2013 demonstrated that at least 552 unique patients would be eligible for the study by viral load and primary care visit criteria. This calculation underestimates the actual pool of eligible patients because it excludes those who lack a viral load measurement within 28 days prior to a primary care visit. Based on trial experience during the first nine months of recruitment, we expect that approximately 10% of patients will be ineligible due to cell phone criteria and another 10% will decline participation. Given a planned accrual period of 21 months, we anticipate that the pool of eligible patients is sufficient to satisfy the target of sample size of 304.

#### Analysis plan

Summary statistics will be used to compare study arms descriptively with respect to baseline socio-demographic characteristics and risk factors for the outcome (e.g. new to clinic vs. poorly retained; newly diagnosed vs. not). In addition, mean duration of study follow-up will be compared by arm, and process measures (e.g. rate of response to Type 2 and 3 messages, self-reported interruptions in cell phone service) will be described.

Following the intention to treat principle, participants will be analyzed in the arms to which they were randomly allocated. Virologic suppression will be analyzed using generalized estimating equation log-binomial models [44],[45] to estimate 12 month mean (95% CI) prevalence by arm and relative risk between arms, adjusted for the stratification factor (newly diagnosed in past 12 months vs. earlier), while accounting for correlation among clustered binary responses at 6 and 12 month follow up. As described in Table 2, virologic suppression status will be missing only if the participant is both lost to the clinic and to the study during the specified data collection window. Model extensions will determine the explanatory effects on virologic suppression of 1) retention in care as a mediator of effect, and 2) intensity of SMS exposure (per protocol analysis).

A log-binomial model will also be used to estimate 12-month mean (95% CI) visit adherence rate by arm and the relative rate between arms, adjusted for the stratification factor and correlation among responses per participant. Like the virologic suppression outcome, the ordinal retention in care outcome will be evaluated for each 6-month study period. For each participant, two indicators per period will be calculated: all scheduled primary care appointments were kept (true/false) and some scheduled primary care appointments were kept (true/false). At the end of the trial, the data will be further reduced to two indicators: all scheduled primary care appointments kept in both periods or some scheduled primary care appointments kept in both periods. Trial-level outcomes will be analyzed using an ordinal logistic regression model to estimate threshold-specific mean (95% CI) odds of retention in HIV care by arm and odds ratios between arms, adjusted for the stratification factor and number of scheduled study visits. If model diagnostics indicate that the proportional odds assumption does not hold, each threshold (all kept vs. fewer than all kept; some/all kept vs. none kept) will be modeled via a logistic regression model. A model extension will analyze study period-level outcomes.

### Additional analyses

#### Cost and cost-effectiveness evaluation

In an era of limited health care resources, a key focus of intervention evaluation is whether the intervention is resource-efficient, thus we plan to investigate the societal economic value of the SMS intervention as well as the economic effect of SMS on the current system of care. Our economic evaluations will test whether the cost of the SMS platform minus any associated savings in downstream costs results in outcomes that are worth this cost. We will conduct two main analyses using welfarist and extra-welfarist approaches: 1) a cost-effective analysis (CEA) of the SMS intervention compared with SMS reminders alone, and; 2) a cost-benefit analysis (CBA) of the intervention with a return on investment (ROI) analysis to the clinic implementing the intervention [46].

CEA determines how to maximize health care effects given the resources available or the health benefit per dollar spent. We will report the incremental cost-effectiveness ratio (ICER) for the SMS intervention compared with SMS appointment reminders alone. Our main outcome will be cost/quality-adjusted life years (QALYs), estimating survival based on patients’ viral load. However, we will also look at differences in cost per differences in meaningful change in virologic suppression. We will use EMR, laboratory, and patient-reported health care utilization data, as well as interviews with the developers and implementers of the SMS intervention and national cost estimates to determine costs. A discount rate of 3% will account for time preference. One-way and probabilistic sensitivity analyses using Monte Carlo simulations will determine the effect of different assumptions on the outcomes of the analysis [47],[48].

Our second economic analysis will be a CBA, which will allow us to determine the economic value of the SMS intervention and SMS appointment reminders alone to the health care system. Cost-benefit analysis determines the intervention with the highest net benefit. Net benefit results can also be ranked for decision-making purposes within a fixed budget [48]. Willingness to pay (WTP) questions will measure benefit. In addition, we will measure the costs of the intervention, any increased or savings in health care expenditures resulting from the intervention and calculate the return on investment of the health care system… This analysis will use the perspective of the health care system and allow intersectoral costs and effects, including non-health related costs.

#### Qualitative sub-study

In order to better understand the experience of the intervention, we will recruit participants for a qualitative sub-study as they finish the 12-month intervention period. We will use one-on-one semi-structured interviews to assess satisfaction with and perceived benefits of the text messages and explore issues related to: 1) barriers to and facilitators of responding to intervention text messages, including access to and comfort with text messaging; 2) frequency and content of text messages, and; 3) privacy and confidentiality. We will also explore the acceptability of other technologies for future interventions.

#### Ancillary studies

We will perform a descriptive analysis of text message communication, including rates of response and reasons for non-response, rates of self-reported cell phone service interruption and reasons for service interruptions, and satisfaction with the amount and content of text messages.

#### Potential harms and human subjects protection

The study was approved by the Committee on Human Research (CHR) at the University of California San Francisco and it has also received a Certificate of Confidentiality from the National Institutes of Health. The greatest potential harm to the study is inadvertent disclosure of HIV status through text messages. However, none of the text messages includes the words HIV or AIDS. Another potential harm is the inconvenience caused by technical glitches, including receipt of messages at an unexpected time, missed messages, or too many messages. There is a possibility that participants may exceed the limit of their text messaging plan and have to pay extra for messages related to the study. Other potential harms include feeling uncomfortable or sad during questionnaire administration or discomfort with phlebotomy. Research assistants receive training to address such risks and carefully review them with participants. Participants are invited to able to ask questions before, during, and following the informed consent process. Adverse events are reported to the study principal investigators and the UCSF CHR using standard protocols.

## Discussion

This study protocol describes a randomized controlled trial designed to improve retention in care and virologic suppression among patients in a U.S. safety-net HIV clinic through an informational, motivational, and supportive text messaging intervention. There are several unique features of the trial that are worth highlighting. First, we sought to develop a text message intervention that could easily be replicated and deployed in a clinic setting without requiring additional staffing, as opposed to other SMS interventions that require in-person follow up of text communication. Second, to increase generalizability, the intervention is clinic-wide, rather than focused on a specific demographic or risk factor sub-group. As such, one of the challenges in designing the intervention was creating text messages that appealed to the clinic population at large. For example, in our formative focus groups, participants who used illicit substances wanted messages about substance use while those who did not use substances or who had been sober for long periods of time did not want any substance use messages. A similar phenomenon was observed with regard to spiritual and religious content. This finding helped us focus the content domains. A third unique feature of the study is that it does not provide cell phones or airtime. While this aspect of the study also increases its generalizability, we acknowledge that cell phone service interruption is common and can reduce intervention exposure. Accordingly, we will supplement the primary intent to treat analysis with per protocol analyses that adjust for intervention exposure. These trial features enable the opportunity to study intervention delivery, as well as associated barriers and costs, in a “real world” context.

One of the key elements in the success of any text messaging trial is partnering with a vendor who can accommodate control and intervention assignments, immediately troubleshoot technical glitches, and securely manage the relationship between phone numbers and de-identified study IDs. The use of pilot participants to test our text messaging protocols allowed us to realize early on that our first vendor did not have the capacity to execute the study as envisioned and to contract with a new vendor.

With regard to study recruitment, it is worth noting that the eligibility criterion of a detectable HIV viral load within 28 days of screening constitutes a rigorous definition of viremia, but that it frequently requires phlebotomy to determine eligibility, posing a potential challenge to recruitment. In addition, recruitment happens in the clinic while enrollment occurs at a geographically distinct research site. Though research assistants offer facilitators such as bus tokens and same-day baseline appointments, inevitably some potential participants fail to present to the research site, even despite reminders. However, we believe that a real strength of our trial lies in this geographic separation, in that it minimizes the possibility that attending study visits will affect the study outcome of clinic appointment attendance.

In sum, we anticipate that findings from our study will provide valuable information about the use of theory-based text messaging to promote retention in HIV care and virologic suppression, further elucidate the challenges of using texting technology with marginalized urban populations, and help inform the development of new mobile health strategies to improve HIV care cascade outcomes.

## Author contributions

KC participated in the design of the study and drafted the manuscript. ER, AC, and JM participated in the design of the study. LW participated in the design of the study and helped draft the manuscript. LC, VF, JA, and JT helped create the recruitment and texting protocols. JH participated in the design of the study, performed the sample size calculation, and helped draft the manuscript. All authors read and approved the final manuscript.

## Authors’ original submitted files for images

Below are the links to the authors’ original submitted files for images. Authors’ original file for figure 1 Authors’ original file for figure 2
