# Supplementary figures and images for: A text messaging intervention to improve retention in care and virologic suppression in a U.S. urban safety-net HIV clinic: study protocol for the Connect4Care (C4C) randomized controlled trial
Source: BMC Infect Dis. 2014 Dec 31;14:718. doi: 10.1186/s12879-014-0718-6 (PMC4323139; doi:10.1186/s12879-014-0718-6)

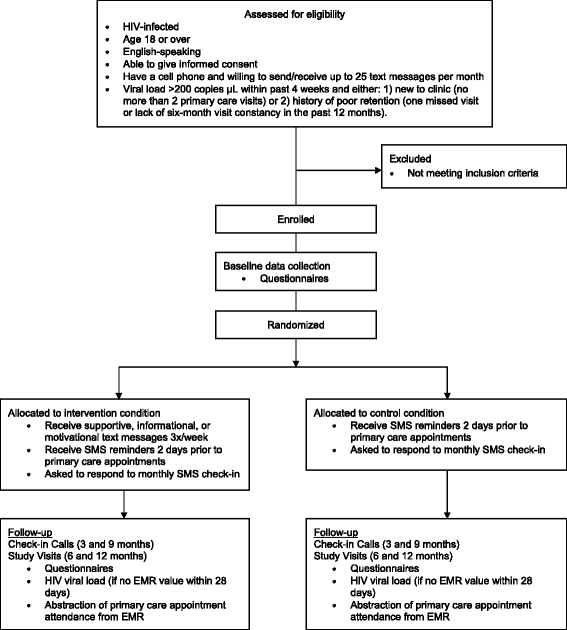

Supplement: Supplementary file 1 — Authors’ original file for figure 1 [file 12879_2014_718_MOESM1_ESM.gif]

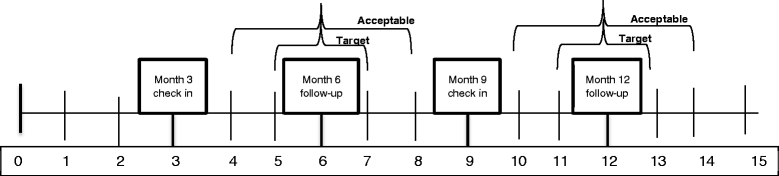

Supplement: Supplementary file 2 — Authors’ original file for figure 2 [file 12879_2014_718_MOESM2_ESM.gif]
